# Cerebral Sinovenous Thrombosis in Neonates and Children

**DOI:** 10.15844/pedneurbriefs-30-3-7

**Published:** 2016-03

**Authors:** Melissa G. Chung

**Affiliations:** 1 Division of Critical Care Medicine and Neurology, Nationwide Children's Hospital, The Ohio State University, Columbus, OH

**Keywords:** Venous Thromboses, Cerebral Thrombosis, Brain Venous Infarction, Neuroimaging

## Abstract

Investigators from Erasmus University Hospital in Belgium and Gustave-Dron Hospital and Roger-Salengro Hospital in France studied the clinical and neuroradiologic characteristics of cerebral sinovenous thrombosis (CSVT) in neonates and children.

Investigators from Erasmus University Hospital in Belgium and Gustave-Dron Hospital and Roger-Salengro Hospital in France studied the clinical and neuroradiologic characteristics of cerebral sinovenous thrombosis (CSVT) in neonates and children. The authors retrospectively reviewed charts for 11 neonates and 16 older children under the age of 18 that were diagnosed with CSVT between 2011 to 2014. Seizures occurred in 26.9% of patients. Systemic illness as a risk factor was more common in the neonates and 36.4% patients did not have a pre-disposing risk factor. Imaging characteristics also differed between the neonatal versus non-neonatal populations. Thrombosis affected the deep venous structures in most of the neonates (51.8%). All neonates had evidence of intraventricular hemorrhage and many also had parenchymal infarcts (38.5%). Extra-parenchymal lesions were more common in neonates than non-neonates and parenchymal lesions tended to be larger in neonates. Cortical vein thrombosis and superficial vein involvement were seen more frequently in the older children (66.7%, p=0.01). This retrospective study provides further information about the clinical presentation and imaging in pediatric CSVT and differences between neonatal and non-neonatal populations. [1]

COMMENTARY. Cerebral sinovenous thrombosis occurs in approximately 0.67 per 100,000 children with a neonatal predominance [2, 3]. The results of this study suggest that the radiological presentation and venous structures involved in CSVT differ between neonates and older children.

The pediatric stroke literature historically has focused upon the superficial cerebral venous system; the superior sagittal sinus (SSS) is reported to be the most frequently involved sinus in pediatric CSVT, especially in neonates [2]. A recent study found that neck positioning and occipital bone compression of the SSS in supine neonates may contribute significantly to the development of neonatal CSVT [3]. In contrast, neonates in this study were more likely to have thrombosis of the deep venous system than of the superficial venous system. The involvement of the deep venous system may explain the high incidence of hemorrhage in neonatal CSVT. The deep venous system drains the germinal matrix so vascular congestion may lead to injury of the germinal matrix (especially if it is immature) and subsequent intraventricular and extra-parenchymal hemorrhage. However, the high prevalence of deep venous thrombosis in this study may have been affected by the small number of neonates (11), all of whom presented with intraventricular hemorrhage.

This study also found a significant portion of older children had cortical vein thrombosis which provides further evidence that cortical vein thrombosis may be underreported. Traditionally, cortical vein thrombosis has been thought to be rare. However, a recent study of cortical vein thrombosis in children reported that almost a quarter of the patients with CSVT actually had cortical vein thrombosis; the children with cortical vein thrombosis also were more likely to have significant neurological complications, such as infarction and seizures [5].

Overall, the results of this study indicate that that thrombosis of the deep venous system and cortical veins may play significant roles in the pediatric CSVT and warrant further investigation. Given the differences in involvement of the venous structures and associated brain injury, this study also provides additional evidence that CSVT in neonates and older children are not a single entity and that studies evaluating etiology, treatment and outcome should differentiate between neonatal and non-neonatal populations.
